# Insights into the precipitation kinetics of CaCO_3_ particles in the presence of polystyrene sulfonate using *in situ* small-angle X-ray scattering

**DOI:** 10.1107/S1600576723005356

**Published:** 2023-07-14

**Authors:** A. Gibaud, D. Younas, L. Matthews, T. Narayanan, K. Longkaew, I. U. Hageberg, Y. Chushkin, D. W. Breiby, B. Chattopadhyay

**Affiliations:** a IMMM, Le Mans Université, Bld O. Messiaen, 72085 Le Mans, Cedex 9, France; b Department of Physics, Norwegian University of Science and Technology, Høgskoleringen 5, Trondheim 7491, Norway; c European Synchrotron Radiation Facility, 71 Avenue des Martyrs, 38043 Grenoble, Cedex 9, France; Australian Centre for Neutron Scattering, ANSTO, Australia

**Keywords:** small-angle X-ray scattering, SAXS, Porod constant, time-resolved studies, calcium carbonate precipitation, polystyrene sulfonate, biomineralization

## Abstract

The formation of calcium carbonate (CaCO_3_) nanoparticles (NPs) in the presence of polystyrene sulfonate (PSS) as an additive was examined by time-resolved small-angle X-ray scattering (SAXS) in a flow system that mimics experimental conditions used at home facilities where the precipitation can be achieved in a beaker. The experiments were carried out at low concentrations to remain in the dilute regime. A model-independent analysis was performed using the Porod invariant which defines the scale factor, leaving only the distribution of radii as the adjustable parameter. The presence of the PSS additive strongly retards the precipitation of CaCO_3_ NPs. The formation of NPs reaches a state of equilibrium after a few minutes. Here, it is shown that the concentration of precursors at a fixed PSS concentration plays a key role in determining the size of the NPs obtained. A full analysis of the SAXS patterns was carried out using the Hurd–Flower model to account for the weaker intensity decay than the classical Porod behaviour. The temporal evolution of the particle radii was determined. Wide-angle X-ray scattering experiments carried out simultaneously show that the particles formed have the structure of vaterite with growth consistent with the evolution of the Porod invariant.

## Abstract 2

The kinetics of precipitation of CaCO_3_ particles in the presence of polystyrene sulfonate was studied by small- and wide-angle X-ray scattering. Employment of the Porod invariant and the Hurd–Flower model provides essential information on the kinetics.

## 1. Introduction

The kinetics of calcium carbonate (CaCO_3_) precipitation are a rather difficult problem to study as they strongly depend on many external factors such as pH, temperature, stirring rate, initial concentration of the precursors and the presence of specific additives (Declet *et al.*, 2016 ▸; Tai & Chen, 1998 ▸). Although CaCO_3_ is ubiquitous in nature and widely used in many industrial applications (Söhnel & Mullin, 1982 ▸; Kang *et al.*, 2020 ▸; Phuhiangpa *et al.*, 2020 ▸), the crystallization of CaCO_3_ remains rather mysterious. In living species, it is achieved by biomineralization, a process in which the intervention of proteins is mandatory. Biomineralization produces remarkable structures such as those encountered in shells, coral, pearls and coccolithophores (Addadi *et al.*, 2006 ▸). It is also through biomineralization that pathological processes can occur, for example formation of kidney stones (Khan *et al.*, 2016 ▸). In these cases, the formation of CaCO_3_ is a slow process, which can be even slower for the growth of stalactites in caves. Conversely, the synthetic precipitation can be fast (<1 s) when Ca^2+^ ions are mixed with carbonate ions in high-supersaturation conditions (*S* = [Ca^2+^][CO_3_^2−^]/Ksp > 10, where Ksp is the solubility product of the polymorph) (Beck & Andreassen, 2010 ▸; Bolze *et al.*, 2002 ▸; Liu *et al.*, 2010 ▸).

CaCO_3_ can precipitate into multiple crystalline polymorphs. Among the anhydrous forms, calcite is the most stable and the two less stable forms are vaterite and aragonite. Of the hydrated forms, two crystal polymorphs, ikaite and mono­hydrocalcite, are stable and can be found in the Earth’s crust (Bush, 1974 ▸). Another hydrated form, calcium carbonate hemihydrate was discovered in 2019 (Zou *et al.*, 2019 ▸). Besides the crystalline forms of CaCO_3_ noted here, various forms of amorphous calcium carbonate (ACC) are also described in the literature. ACC is considered to be a thermodynamically unstable precursor phase that forms during the first crystallization events and is consumed during the precipitation of the previously mentioned stable forms of CaCO_3_ (Pontoni *et al.*, 2003 ▸; Ševčík *et al.*, 2015 ▸). Although the pathways for its formation are not very well defined, ACC is shown to convert to vaterite at low temperatures and then yield calcite or aragonite at higher temperatures (Rodríguez-Blanco *et al.*, 2011 ▸).

ACC in combination with organic macromolecules and inorganic ions is considered to be the chosen pathway for micro-organisms to form larger crystals without any need of either high-temperature melt phases or high-supersaturation solution conditions, which makes it more intriguing to study (Weiner *et al.*, 2005 ▸). Among the polymorphs of calcium carbonate, vaterite is found to be the most suited for applications in, for example, biomedicine (Hassani *et al.*, 2013 ▸) because of the multitude of structural hierarchies that can be engineered due to it porous structure. It usually precipitates in the form of spheroidal particles (Chavez Panduro *et al.*, 2012 ▸; Cherkas *et al.*, 2017 ▸). Aragonite, on the other hand, appears at temperatures above 60°C (Ogino *et al.*, 1987 ▸) and usually precipitates in the form of nanorods, which agglomerate into bundles.

Many studies have been carried out to monitor the early stages of the kinetics of CaCO_3_ precipitation with various techniques (Dreybrodt *et al.*, 1997 ▸; Bolze *et al.*, 2002 ▸; Gebauer *et al.* 2008 ▸, to cite a few). It has been shown that the precipitation rate of CaCO_3_ from supersaturated solutions in the H_2_O, CO_2_, CaCO_3_ system is controlled by three rate-determining processes: the kinetics of precipitation at the mineral surface, the mass transport of the reaction species involved and the slow kinetics of the overall reaction, HCO_3_^−^ + H^+^ → CO_2_ + H_2_O (Dreybrodt *et al.*, 1997 ▸). Conductivity experiments in a controlled CO_2_ atmosphere carried out for calcite precipitation highlighted the critical role played by the slow reaction (Dreybrodt *et al.* 1997 ▸). However, conductivity measurements do not provide any structural information about the particles, for instance, their size, shape, density and formation pathway.

Bolze *et al.* (2002 ▸) demonstrated the use of rapid stopped-flow mixing in combination with small-angle X-ray scattering (SAXS) which facilitated probing morphological features in the early stages of precipitation. In their work they obtained comprehensive information about the size modification, mass density, number density and aggregation behaviour of the CaCO_3_ particles formed as a function of the reaction time. Evidence for the formation of ACC, a metastable precursor of calcite, was observed. It was shown that for a 9 m*M* initial salt concentration of (CaCl_2_ and Na_2_CO_3_) precursors the reaction was over after 100 s, leading to the formation of amorphous particles of 133 nm radius, having a relatively low polydispersity. The growth in this case was clearly measurable after 0.5 s, with the particles having a radius of 32 nm. Gebauer *et al.* (2008 ▸) have shown that at fixed pH very small stable clusters (2–6 nm in diameter) exist before nucleation begins. Once a critical cluster concentration is reached, the nucleation takes place and ACC is formed.

As a general rule, it can be stated that the precipitation of CaCO_3_ particles largely depends on the reaction parameters such as temperature, pH and concentration of reactants (Declet *et al.*, 2016 ▸; Tai & Chen, 1998 ▸). Whether a specific polymorph is formed after the appearance of ACC remains unclear. It becomes more complex to identify which polymorph will form when the ionic strength of the initial solutions is modified by the addition of specific ions such as Na^+^ and Mg^2+^. Of particular interest is the development of strategies to stabilize a specific polymorph. Indeed, many living creatures select different polymorphs for specific functions. Aragonite is found in nacre, while mussels use aragonite in the inner part of their shells and calcite in their outer part. In some cases, ACC plays a crucial role in the structuration of shells (Grünewald *et al.*, 2022 ▸; Weiss *et al.*, 2002 ▸). The role of confinement to favour the growth of aragonite in submicrometre pores in the absence of any additives was shown by Zeng *et al.* (2018 ▸). These studies suggest that organisms exploit confinement effects to control polymorphism.

Different methods have been adopted to avoid the transformation of vaterite into calcite by the dissolution–recrystallization process. In particular, additives acting as stabilizing agents are clearly efficient in promoting the formation of mesocrystals (Cölfen & Antonietti, 2005 ▸; Didymus *et al.*, 1993 ▸; Gehrke *et al.*, 2005 ▸; Geng *et al.*, 2010 ▸; Isopescu *et al.*, 2010 ▸; Kulak *et al.*, 2007 ▸; Zhu *et al.*, 2009 ▸).

Polystyrene sulfonate (PSS), a polyelectrolyte, has been identified as a potent additive to stabilize vaterite (Wang *et al.*, 2005 ▸; Cai *et al.*, 2008 ▸; Smeets *et al.*, 2015 ▸). The sulfonate groups of PSS bind well to Ca^2+^ ions and prevent the rapid precipitation of CaCO_3_ particles. The PSS molecules are then embedded inside the particles, preventing their transformation into calcite via the dissolution–recrystallization process, even if the particles are kept in water for several hours at *T* = 75°C (Beuvier *et al.*, 2022 ▸). It was also shown that, depending on the initial concentration of the precursors, core–shell particles can be formed. Beyond 24 h, hollow-core vaterite particles stabilized with PSS are obtained.

Although the formation of vaterite particles using PSS as an additive has been well documented in the literature (Wang *et al.*, 2005 ▸; Cai *et al.*, 2008 ▸; Smeets *et al.*, 2015 ▸; Beuvier *et al.*, 2022 ▸), there is not much information available on the formation kinetics of such particles because the precipitation process is relatively rapid [less than a few minutes as reported by Beuvier *et al.* (2022 ▸)]. The focus of the present investigation is a systematic time-resolved SAXS study of CaCO_3_ precipitation for different concentrations of the initial precursors in the presence of a fixed PSS concentration. We consider it likely that the confined geometry of stopped-flow devices might modify the particle morphology and reaction kinetics of the vaterite–PSS complexes. Hence, in this study we chose to carry out the mixing in a beaker rather than a stopped-flow device. This enabled us to address reaction kinetics under conditions similar to those that we have used and reported before (Beuvier *et al.*, 2022 ▸), where the mixture of the initial precursors in a beaker was performed by stirring instead of in the confined geometry of the stopped-flow device.

## 2. Experimental

The two precursors, CaCl_2_·2H_2_O (*M*_w_ = 147 g mol^−1^) and Na_2_CO_3_ (*M*_w_ = 106 g mol^−1^), and poly(4-styrene sulfonate) (PSS; *M*_w_ = 70000 g mol^−1^) were purchased from Aldrich and were used as purchased. Two solutions of the initial precursors were prepared at concentrations *c* = 9.0, 7.0 and 4.5 m*M* in MilliQ water (18.2 MΩ cm). A mass of 50 mg of PSS was added to the CaCl_2_ precursor solution to stabilize the formation of vaterite during the precipitation and to slow down the kinetics of formation by complexation with the Ca^2+^ ions.

The formation of CaCO_3_ particles by precipitation of the precursors was monitored by SAXS at the ID02 beamline of the European Synchrotron Radiation Facility (ESRF), Grenoble, France (Narayanan *et al.*, 2022 ▸). The X-ray energy was 12.23 keV (λ = 0.101 nm) with a single sample-to-detector distance of 10 m covering a *q* range of 6 × 10^−3^–7 × 10^−1^ nm^−1^, where *q* is the magnitude of the scattering vector, given by *q* = (4π/λ)sin*θ* with the scattering angle 2θ. The initial precursors were mixed under stirring at a rate of 500 r min^−1^ at *t* = 0 s in a beaker and pumped through a quartz capillary (ø = 2 mm) using a small peristaltic pump (see the experimental setup in S1 of the supporting information). The precipitation was monitored as a function of time using an acquisition sequence where frames were taken every second whilst the solution was flowing at a rate of 200 µl s^−1^. Measurements began with a constant time delay of *t* = 60 ± 5 s due to the time necessary for interlocking the experimental hutch. 2D SAXS data were collected using an Eiger2 4M (Dectris) pixel array detector and the sample transmission was simultaneously measured. The acquired 2D patterns were normalized to an absolute intensity scale and azimuthally averaged to obtain the 1D SAXS profiles. Then, the corresponding normalized background (the water-filled quartz capillary) was subtracted from the 1D profiles. Wide-angle X-ray scattering (WAXS) data were collected simultaneously using a Rayonix LX170-HS CCD detector at a sample-to-detector distance of 0.126 m, covering the *q* range 5.9–44.3 nm^−1^.

The morphology of dried CaCO_3_ particles was imaged using a JEOL JSM 65VL scanning electron microscope (SEM) operated at 20 kV with a working distance of 11 mm.

## 3. Small-angle scattering theory

In the case of nanoparticle (NP) formation, one can start the small-angle scattering (SAS) analysis with the scattering of spherical particles. The scattering amplitude for a uniform sphere in a solvent is given by the Fourier transform of the electron density contrast ρ − ρ_Sol_ between the particle and the solvent, which is constant within the radius *R*. SAS theory is thoroughly covered in the literature (Deschamps & De Geuser, 2011 ▸; Glatter, 1977 ▸, 1982 ▸; Guinier & Fournet, 1955 ▸).

For a distribution of *N* independent spheres of the same size, the scattered intensity (see S2 of the supporting information) is given by

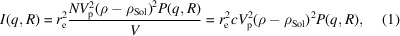

where *c* is the particle concentration, *V* and *V*_p_ are the irradiated volume and the volume of the particle, respectively, and *r*_e_ = 2.8 × 10^−15^ m is the classical radius of an electron. The term *P*(*q*, *R*) is the normalized scattering form factor of the spherical object [see equation (1) and Fig. S2 of the supporting information]. Equation (1) has the dimension of an inverse distance and is usually expressed in cm^−1^ or mm^−1^. It can be directly compared with the experimental measurement provided that the data have been normalized to absolute units.

It immediately follows from equation (1) that the intensity strongly depends on the volume of the particles and on the concentration. As *I*(*q*, *R*) scales with *R* ^6^, when large particles are present in a solution, they tend to dominate the scattering at low *q*, submerging the contribution of smaller ones.

For polydisperse independent particles with a size distribution function *f*(*r*), the scattered intensity is defined as

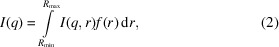

where *R*_min_ and *R*_max_ are values under and above which *f*(*r*) = 0. Typically we can consider these values to be defined as *R* ± 3σ, where *R* is the mean value of the radius and σ is the standard deviation of the distribution. Gaussian, Schultz and lognormal distributions are usually used to represent the polydispersity. An example of a Gaussian distribution of radii with σ = 15 nm for a sphere of radius 150 nm is provided in Fig. S2(*a*). It can be seen that the effect of polydispersity is to damp the oscillations of the normalized intensity. Note that the high *q* range is asymptotic to *q* ^−4^. In the following we will mainly use a lognormal distribution (Hosking & Wallis, 1997 ▸).

Another way to investigate how a solution of particles scatters is to use Kratky plots, which are obtained by plotting *I*(*q*)*q* ^2^ versus log(*q*). An example of a Kratky plot for a sphere of 150 nm radius and σ = 0 and 15 nm is shown in Fig. S2(*b*).

One can see that *I*(*q*)*q* ^2^ shows a maximum intensity at the position *q*_max_ related to the Guinier radius (Deschamps & De Geuser, 2011 ▸; Guinier & Fournet, 1955 ▸),

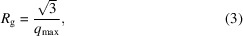

and corresponds for monodisperse spherical objects to a sphere of radius *R*_sphere_, where

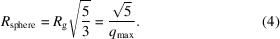

Note that the maximum for σ = 15 nm in Fig. S2(*b*) is slightly shifted towards smaller *q* values as a result of a higher contribution of larger particles. In addition, we observe a slight discrepancy between the input value of *R* and that determined by the measurement of *q*_max_ even for monodisperse particles. This discrepancy signifies that equation (4) calculates an approximate radius but should not be taken for granted as an exact value.

Furthermore, from the Kratky plot (Glatter *et al.*, 1982 ▸), one can derive the Porod invariant *Q*_Porod_, given by

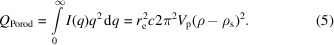

In this specific case above, where it is assumed for simplicity that 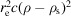 = 1 nm^−7^, the numerically calculated value of the Porod invariant was *Q*_Porod_ = 2.79 × 10^8^ mm^−1^ nm^−3^ using equation (5), with *Q*_Porod_ = 2.64 × 10^8^ mm^−1^ nm^−3^ for σ = 0 nm and *Q*_Porod_ = 2.7 × 10^8^ mm^−1^ nm^−3^ for σ = 15 nm, calculated using numerical integration carried out by the trapezoidal method. Note that, for an absolute measurement where intensity is measured in mm^−1^, *Q*_porod_ will be expressed in mm^−1^ nm^−3^. The agreement is fairly good given the fact that the integration is performed up to *q =* 0.2 nm^−1^. There is very little difference (∼5%) between the three values of *Q*_Porod_ despite a 10% polydispersity having been introduced in the case of polydisperse particles. The determination of the Porod invariant requires the measurement of the scattered intensity at a *q* value which is sufficiently small to properly show the maximum. Otherwise, the Porod invariant will be underestimated, and a scale factor needs to be introduced to adjust the calculated intensity to the observed one. On the other hand, the high-*q* dataset contributes far less to the Porod invariant in the specific case, where the intensity *I*(*q*) falls as *q* ^−4^.

A combination of the Porod invariant together with equations (1) and (2) yields

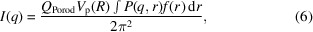

where *R* is the mean value of the distribution of radii.

Equation (6) is particularly interesting as it shows that the scattered intensity scales as *Q*_Porod_ *V*_p_(*R*)/(2π^2^) which has units of mm^−1^. Then, the only unknown in the calculation of a dilute solution of particles, for the determination of the absolute intensity, is the size distribution of the particles once the Porod invariant has been calculated. Note that the Porod invariant used as a scale factor depends on several parameters, namely the particle concentration, the volume of the particles and the contrast of electron density [see equation (5)]. A fit to the data allows an estimation of the radius distribution of the particles and therefore their volume, yet the concentration and the contrast of electron density remain correlated. The contrast of electron density is in particular strongly dependent on the porosity of the particles and on their structural composition. The use of the Porod invariant makes it possible to circumvent this bias. Note that the Porod invariant is usually used as a way to determine the volume of the particles after extrapolation of the intensity at *q* = 0 (Liu *et al.*, 2010 ▸).

Another constant known as the Porod constant is obtained by calculating the limit of *I*(*q*)*q* ^4^ at high *q* [see Fig. S2(*c*)]. For spherical particles with a smooth surface, it is expected that the intensity will fall off as 1/*q* ^4^ so that *I*(*q*)*q* ^4^ should reach a constant value at high *q*. This limit provides essential information on the surface *S* of the particles via the following relationship:

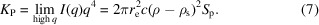

In this specific example, we obtain a theoretical value *K*_P_ = 1.77 × 10^6^ mm^−1^ nm^−4^ using equation (7) in which 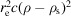 = 1 nm^−7^ and *K*_P_ = 1.79 × 10^6^ mm^−1^ nm^−4^ in the case of σ = 15 nm from the graph shown in Fig. S2(*c*). For σ = 0 nm, the oscillations are not damped and *K*_P_ cannot be determined graphically. It is also clear from equations (5) and (7) that the ratio *S*_p_/*V*_p_ is given by

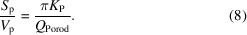

In the chosen example after numerical integration, *S*_p_/*V*_p_ = 0.02 nm^−1^, which is equal to 3/*R*.

In the case where the scattering by spherical particles does not fall as 1/*q* ^4^, it is possible to use different models among which one can find the mass and surface fractal models. Fractals are self-similar over a certain spatial range. When building a model to describe experimental fractals, it is necessary to define a spatial limit which is achieved using a cut-off function.

Here we have used the fractal model developed by Hurd & Flower (1988 ▸) which is appropriate to describe particles approaching spherical geometry (Bale & Schmidt, 1984 ▸; Hurd & Flower, 1988 ▸; Schaefer & Hurd, 1990 ▸). Initially, it was used to interpret the growth of pyrolytic silica aggregates. Here, it is shown to be effective in describing CaCO_3_ particles in solution because the scattering data in this study were not decaying perfectly as 1/*q* ^4^. The form of the cut-off function *h*(*r*) of a perfect homogeneous sphere is defined by the degree of autocorrelation (Sorbier *et al.*, 2019 ▸; Thissen & Brandon, 2015 ▸; Torquato, 2002 ▸) as

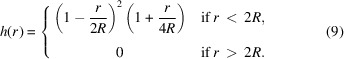

The scattered intensity in this model is given by

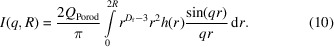

When the fractal dimension *D*_f_ = 3, equations (6) and (10) are strictly equivalent. For *D* < 3, we empirically found that, with our data, equation (10) needs to be renormalized by a factor roughly equal to *A*/*R* ^(D_f_^ ^−3)^. *A* is a scale factor that is equal to 1 when the measurements are carried out at a low value of *q*, where any underestimation of the Porod invariant is avoided. Note that for fractal objects, *D*_f_ can be a non-integer, or fractional, and can vary between 1 and 3 for an object embedded in three-dimensional space. Qualitatively, the object is uniformly dense when *D*_f_ = 3 and becomes increasingly open and ramified as *D*_f_ decreases (Schaefer & Hurd, 1990 ▸). Use of the Hurd–Flower model seems to be quite appropriate to deal with particles such as vaterite particles [shown to be porous by Chavez Panduro *et al.* (2012 ▸) and Cherkas *et al.* (2017 ▸, 2018 ▸)]. It is shown in these latter studies that the vaterite particles are not uniformly dense and are made up of aggregated nanodomains responsible for the broadening of the Bragg reflections in the X-ray powder diffraction data. As we will show in the next section, since the observed scattering does not follow a 1/*q* ^4^ decay, it is mandatory to use a model like the Hurd–Flower to properly describe the datasets. A full discussion of the effect of fractality in SAXS analysis can be found in the literature (Hurd & Flower, 1988 ▸; Schaefer *et al.*, 1987 ▸).

## 4. Results and discussion

### 4.1. On the precipitation of the 9.0 m*M* concentration

**Figure 1:**
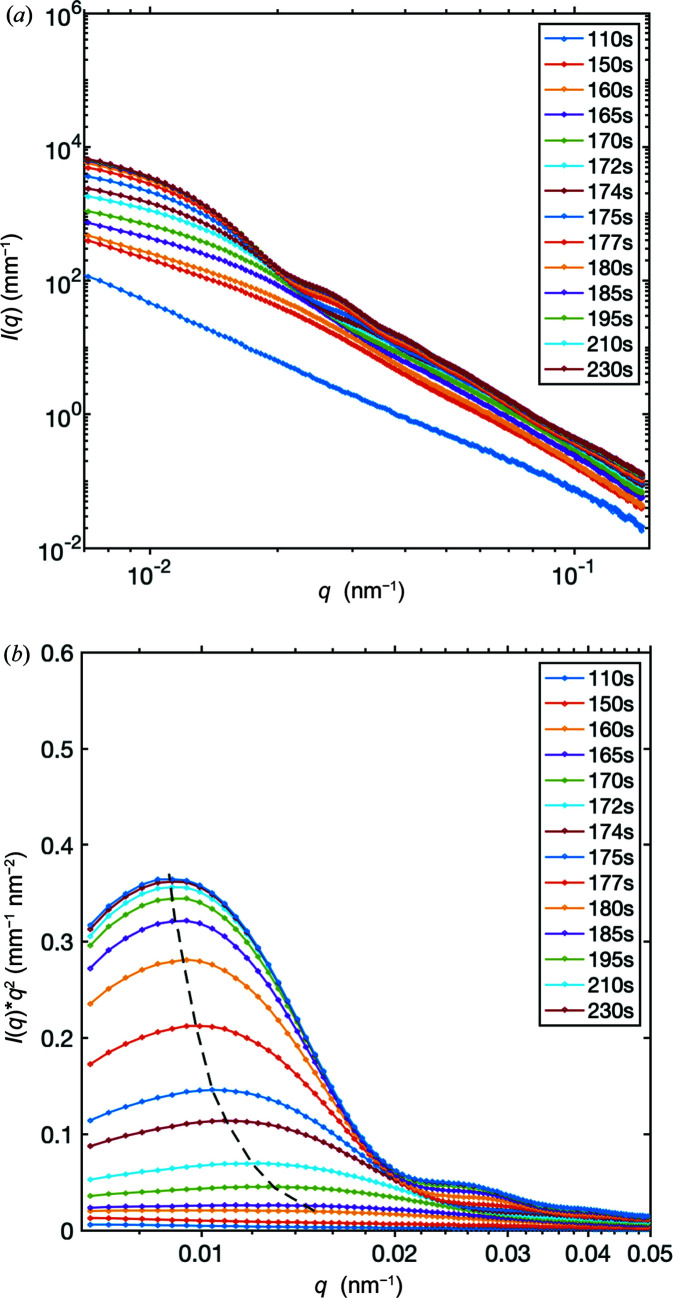
1D SAXS profiles of the CaCO_3_ precipitation process during flow experiments as a function of time. (*a*) Logarithmic plot of the scattered intensity. (*b*) Kratky plot of the scattered intensity, where the dashed line is a guide to the eye to show the location of the maximum in the Kratky plot.

Fig. 1 ▸(*a*) shows the evolution of the SAXS profiles during an experiment where CaCl_2_ and PSS were mixed with Na_2_CO_3_. It is evident from Fig. 1 ▸(*a*) that the scattering at time *t* = 110 s after mixing does not show many features except an almost linear decay in the logarithmic plot. With elapse of time, the scattering profile increasingly develops some clear oscillations. Between *t* = 150 s and *t* = 175 s, the oscillations are very broad and of low amplitude. For *t* > 175 s, the number of fringes increases but they are clearly damped at high *q* values (*q* > 6 × 10^−2^ nm^−1^). During the whole experiment, the intensity at the lowest *q* (*q* ≃ 7 × 10^−3^ nm^−1^) increases significantly. At *t* = 210 s and upwards, there is no further evolution of the scattering profile, demonstrating that the system has reached a terminal state.

All the above features can be interpreted as resulting from the formation of independent spherical polydisperse NPs, first undergoing growth before reaching a maximum radius. The initial power law decay before the nucleation of particles is attributed to the aggregates of PSS. Additionally, the number of particles, indicated by the intensity at low *q*, also reaches a maximum value that does not change after *t* > 210 s. Therefore, we conclude that, in the presence of PSS and after 230 s, we have reached a terminal state, after which the size of particles does not change. The Kratky plot [Fig. 1 ▸(*b*)] exhibits a maximum where the *q* position, *q*_peak_, is shifted towards lower *q* as *t* increases, showing the growth of particles as a function of time. The intensity of the maximum also increases due to the increasing size of the particles in the solution.

**Figure 2:**
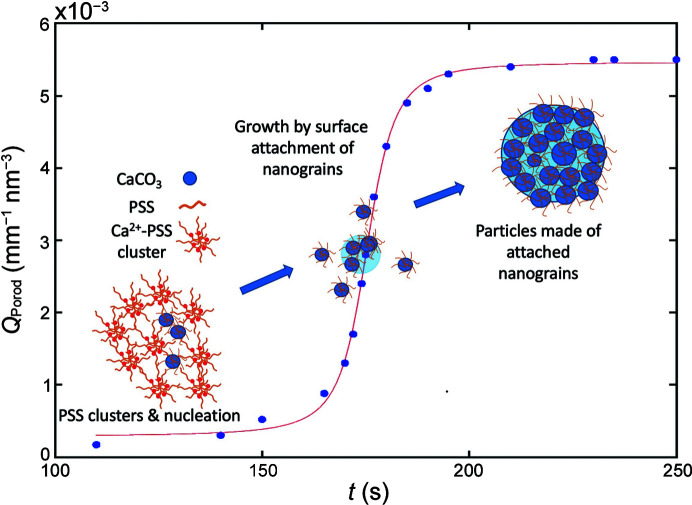
Evolution of the Porod invariant as a function of time. Blue data points are the observed Porod invariant, and the red solid line is calculated according to the algebraic function, equation (11).

Another way to probe how the system is evolving is to determine the time dependence of the Porod invariant as depicted in Fig. 2 ▸. An improved estimation of the Porod invariant *Q*_Porod_ was made in this case, by extrapolating the data over a larger *q* range than the measured dataset (0.2 > *q* > 0.001 nm^−1^).

One can see that the Porod invariant steeply rises above *t* = 160 s to finally reach a plateau above *t* = 200 s. This evolution can be described by an algebraic function of the sigmoid family defined as

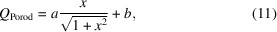

where *x* = (*t* − *t*_c_)/τ, *t*_c_ corresponds to the central time of the sigmoid and 2τ is the characteristic time necessary to finalize the growth by surface attachment shown in Fig. 2 ▸.

**Figure 3:**
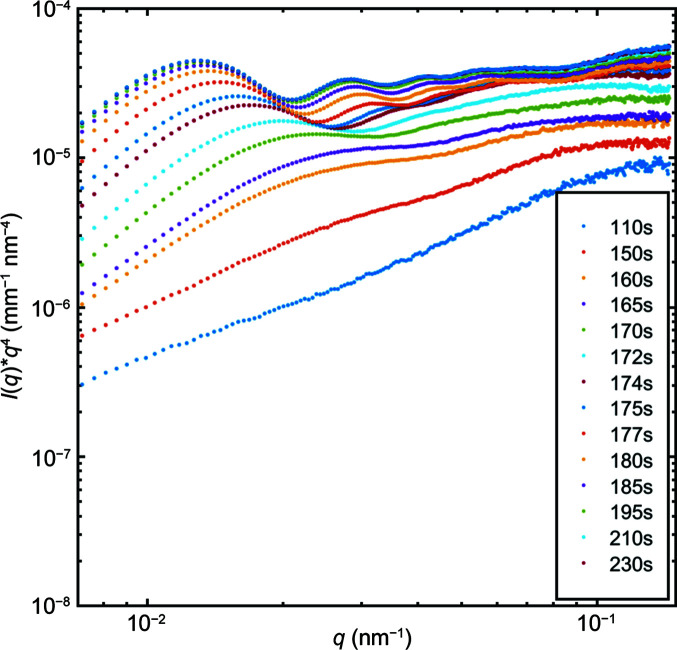
1D SAXS profiles plotted as *I*(*q*)*q* ^4^ versus *q*, demonstrating that at high *q* the slope of the scattering is positive and not constant. This highlights that the scattering decays less rapidly than 1/*q* ^4^.

From fitting of the Porod invariant curve (Fig. 3 ▸), the optimum kinetic parameters obtained were *t*_c_ = 175.3 s, τ = 7.2 s, *a* = 2.6 × 10^−3^ mm^−1^ nm^−3^ and *b* = 2.9 × 10^−3^ mm^−1^ nm^−3^. It can thus be concluded that the formation of particles reaches its maximum at *t* ≃ 200 s after the mixing within a time constant of about 2τ = 14.4 s. This time constant corresponds to the time necessary for the system to move from the presence of nanograins in the solution to larger agglomerated particles. At the end of the process, the Porod invariant reaches a maximum value *Q*_Porodmax_ = 5.4 × 10^−3^ mm^−1^ nm^−3^. The fact that the Porod invariant reaches a plateau shows that the precipitation reaction is nearly finished after about 200 s, indicating that both the number of particles and their size have reached a steady state. Note that, in the absence of PSS, the precipitation of micrometre-sized particles at room temperature occurs on a timescale of a second, which is two orders of magnitude faster [see Figure S7 of Beuvier *et al.* (2022 ▸)]. The delay in the precipitation of CaCO_3_ observed in this experiment can be attributed to the presence of PSS.

Fig. 3 ▸ presents a plot of *I*(*q*)*q* ^4^ versus *q*, where it is apparent that the high-*q* limit is not constant but shows a positive slope. This emphasizes the fact that a homogeneous spherical model cannot fully describe the data and a fractal model is required to analyse the datasets. In the following, we have used both the Hurd–Flower model above *t* > 170 s and a model in which we have added a fractal component *A*/*q* ^p^ to a polydisperse model of spherical particles below (*A* being a scale factor and *p* a fractal exponent). This additive fractal component is assigned to scattering from the aggregates of PSS. The presence of a broad hump at *t* < 175 s with a maximum close to *q* = 0.08 nm^−1^ was modelled using equation (10) with polydisperse spherical particles.

**Figure 4:**
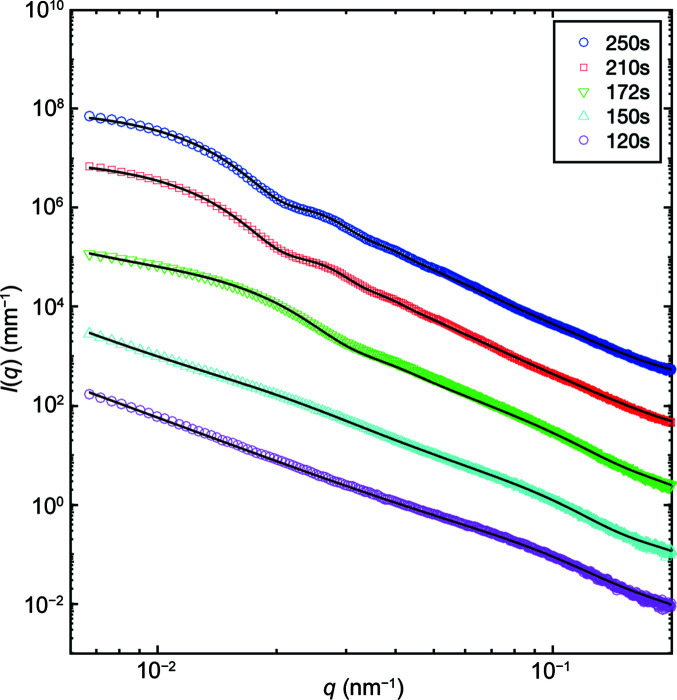
1D fitted SAXS profiles of the CaCO_3_ precipitates at different times using the Hurd–Flower model with a lognormal size distribution of spheres. The datasets are offset by one order of magnitude for clarity (solid lines are calculated values). The lowest curve (magenta circles) is without offset.

Fig. 4 ▸ shows the result of the Hurd–Flower model fitted to our experimental datasets. This model describes the scattering features well and, in particular, faithfully reproduces the high-*q* decay of the intensity. We found that a fractal dimension *D*_f_ = 2.955 adequately describes the data. Although this value is close to *D*_f_ = 3, it is impossible to describe the data with polydisperse spheres of smooth surfaces. The best fit was obtained using a lognormal distribution of radii centred at *R* = 215 nm.

The fits to the data using the Hurd–Flower model are generally good (*t* > 170 s). For *t* < 170 s, the Hurd–Flower model does not agree with the measured data. The disagreement is attributed to the fact that the intensity at small *q* wavevector transfers (*q* < 0.05 nm^−1^) does not reach a plateau as is the case at longer times (*t* > 170 s). The low-*q* upturn can be described by a power-law decay of the intensity as *A*/*q^p^* where *A* is a scale factor and the exponent *p* = 3.2. This would correspond to a surface fractal morphology with exponent *D*_S_ = 6 − *p* = 2.8. Note that PSS is a polyelectrolyte, which under low ionic strength may form complexes with surface fractal morphology (Takahashi *et al.*, 2017 ▸). The hump at *q* = 0.08 nm^−1^ arises from polydisperse spheres. The intensity is thus described by the sum of these two contributions.

### 4.2. Effect of the initial concentration of the precursors

**Figure 5:**
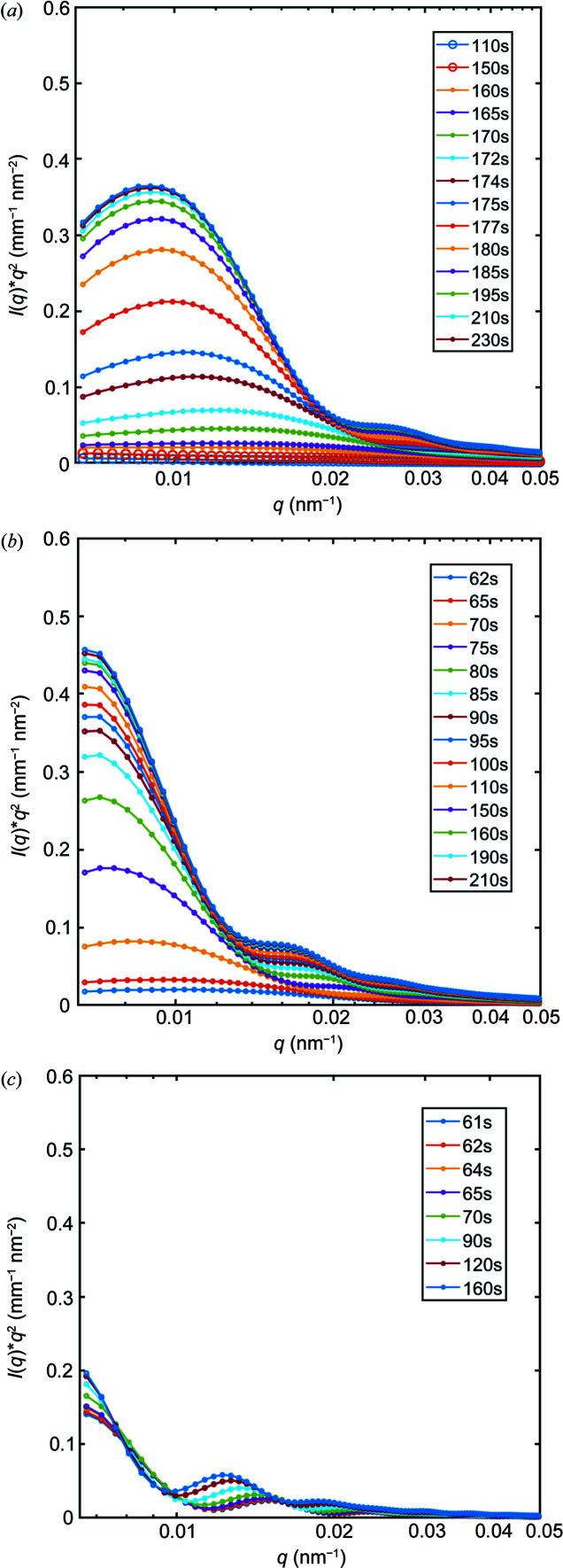
1D SAXS profiles displayed as Kratky plots during the CaCO_3_ precipitation, for precursor concentrations of (*a*) *c* = 9.0 m*M*, (*b*) *c* = 7.0 m*M* and (*c*) *c* = 4.5 m*M*.

The structural evolution of CaCO_3_ was also studied for samples prepared at two other initial precursor concentrations, *c* = 7.0 m*M* and 4.5 m*M*, where the CaCl_2_ solution contained PSS. The Kratky plots shown in Fig. 5 ▸ demonstrate that the maximum of the curve shifts towards lower *q* when the initial concentration of the precursor is decreased, signifying the formation of larger particles. In addition, it can be seen that the kinetics of formation are rapid, as the form factor oscillations can be observed at earlier time frames in the lower-concentration cases. At *c* = 4.5 m*M*, it was not possible to access the early stages of the precipitation because of the mentioned 60 s time delay necessary to start the measurements after mixing the precursors manually. In this low-concentration case, the amplitude of the oscillations is more pronounced, indicating a lower polydispersity of the particles.

**Figure 6:**
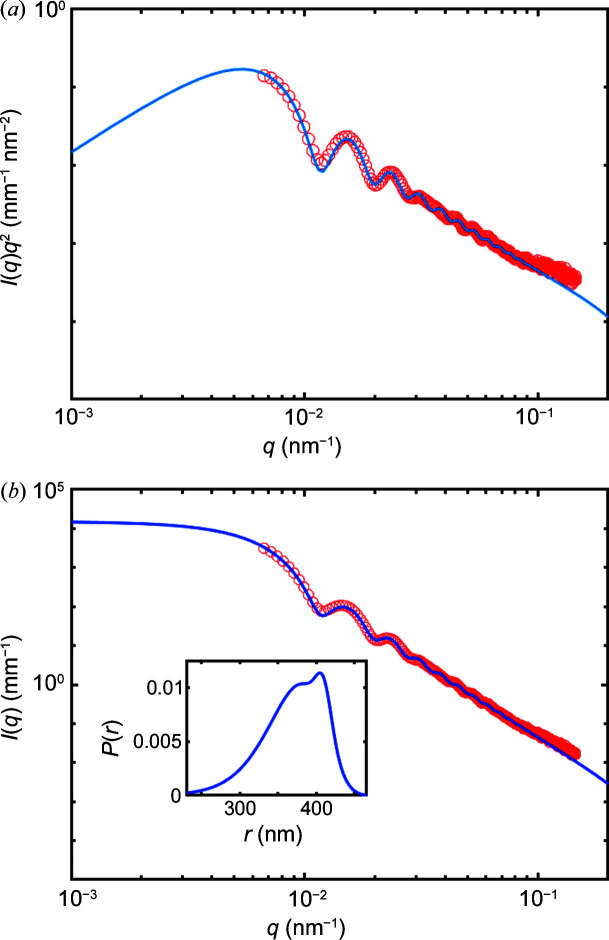
1D SAXS profiles of CaCO_3_ precipitation for *c* = 4.5 m*M* at *t* = 61 s (data points in open circles), with the calculated model using equation (10) (full line). Data displayed as (*a*) a Kratky plot and (*b*) a logarithmic *I*(*q*) versus *q* plot; the inset is the lognormal size distribution to which a Gaussian distribution was added to account for the bimodal distribution.

A fit to the Hurd–Flower model was carried out for all samples in order to determine the evolution of the particle size during precipitation. Note that for *c* = 7.0 m*M* and *c* = 4.5 m*M* it was not possible to determine the Porod constant from the experimental datasets as the first peak was not fully measured [Figs. 5 ▸(*b*) and 5 ▸(*c*)]. In order to obtain the Porod invariant, we simulated the Kratky plot over a larger *q* range starting at *q* = 0.001 nm^−1^ (see Fig. 6 ▸).

**Figure 7:**
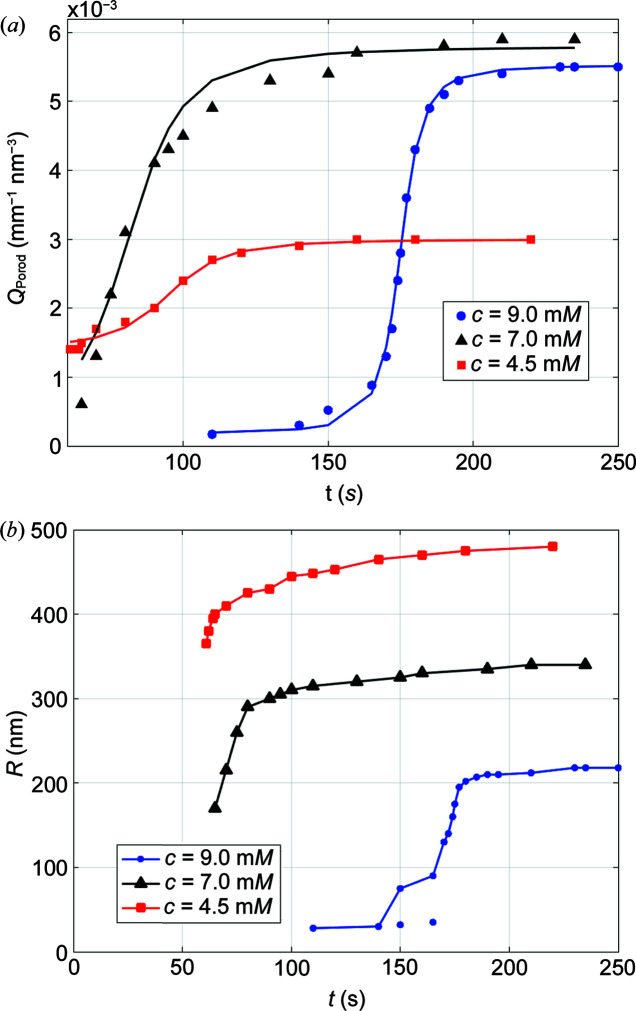
(*a*) The Porod invariant, *Q*_Porod_, as a function of time for the three precursor concentrations [solid lines in this figure are calculated values according to equation (11)]. (*b*) Particle radius, *R*, as a function of time obtained from the Hurd–Flower fits for the three precursor concentrations.

The time evolution of the Porod invariant for different concentrations is plotted in Fig. 7 ▸. One can see that the only concentration for which the full growth was observed is 9.0 m*M*. For the two lower concentrations, the reaction kinetics were much faster, and after 60 s the formation of large particles had already begun. Note that although the particles are larger for *c* = 4.5 m*M*, the Porod invariant remains smaller after saturation, which indicates that the number of particles is much smaller. An intermediate situation is observed for *c* = 7.0 m*M*, where the particles are larger than those for *c* = 9.0 m*M* and the saturation of the Porod invariant is almost equivalent. Again, this is an indication that the concentration of particles increases with the concentration of the precursors.

The Hurd–Flower fit to the data allows the determination of the particle radius during the precipitation of CaCO_3_. One can see that the smaller the concentration, the larger the particles, which can be understood in terms of the number of nuclei, where a higher concentration leads to a larger number of nuclei. In the presence of PSS, the nuclei are stabilized by the affinity of Ca^2+^ ions with the sulfonate groups. When the number of nuclei is large, the growth of each nucleus is limited and the size of the particles is smaller.

It is also clear that at a fixed PSS concentration the number of PSS molecules binding to calcium ions decreases at a higher concentration of the calcium carbonate precursor. This is an additional reason why the particles formed at a lower concentration of the Ca^2+^ precursor are larger since the PSS molecules occupy proportionally more volume in the nanograins and in the final particles (Beuvier *et al.*, 2022 ▸). This could also explain why the kinetics are faster at low Ca^2+^ precursor concentration if the threshold for the growth burst depends on the size of the nanograins.

Note that in the case of *c* = 9.0 m*M* two regimes are observed before the steep change of radii. During the early stages, particles with a radius close to 30 nm are formed [as indicated in Fig. 7 ▸(*b*)]. Their number then grows and between *t* = 150 s and *t* = 172 s a superposition of populations with 25 nm particles and larger ones (*R* > 70 nm) was observed. This observation was not possible for the two other concentrations because of the 60 s time delay to start the measurements.

The presence of two populations of particles is in good agreement with the idea of the work by Gebauer *et al.* (2008 ▸) concerning prenucleation in CaCO_3_ clusters of 2–6 nm in diameter. In our case, the size of these clusters is quite large (*R* ≃ 30 nm for *c* = 9.0 m*M*) so that we can consider them as pre-attached clusters. Smaller clusters pre-exist for up to 170 s and suddenly grow in bursts when *t* > 150 s, as shown by the blue curve in Fig. 7 ▸(*b*). Note that the jump observed in the Porod invariant is concomitant with that observed in the radius of the particles. One could therefore conclude that the large increase in the Porod invariant should be related to the large increase in the radius of the particles. However, the ratio of final values to initial values of *Q*_Porod_ for *c* = 9.0 m*M* is only 32.3 while the change in volume ratio is 383.7. Using equation (5), it can be concluded that the particle concentration must decrease sharply during the jump. This observation is in agreement with the idea of small nanograins agglomerated by surface fixation during the jump. Assuming that the density contrast remains the same before and after the jump, it can be concluded that the particle concentration is about 12 times smaller after the jump. This is probably an underestimated value because the agglomerated particles are known to be porous due to the presence of voids between the attached nanograins.

### 4.3. Wide-angle X-ray scattering

**Figure 8:**
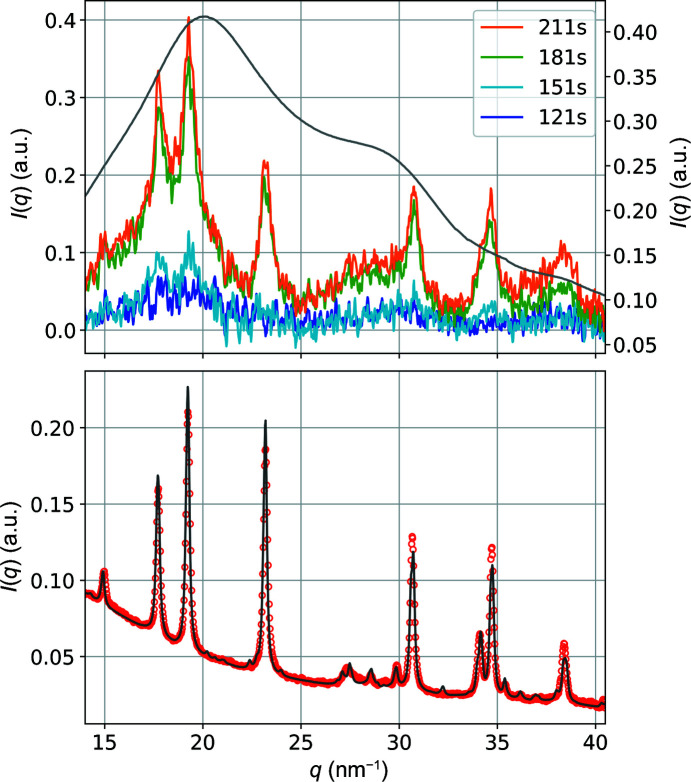
1D WAXS profiles of CaCO_3_ particles obtained during the flow experiment at 9 m*M* concentration. Top panel: particles with water in the capillary (grey line) and after subtraction of the water capillary at different times showing the increase in the peak intensities of the vaterite particles (for clarity, the intensity of the WAXS data after subtraction of the background has been multiplied by a factor 10^5^). Bottom panel: powder coming from the drying of the aliquot after centrifugation (circles are the observed data, the line is the calculated data using the *MAUD* software). In the *C*2/*c* space group, the first three intense peaks correspond to superimposed reflections with Miller indices 002 & 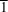 12, 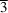 11 & 002 & 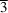 12, and 400 & 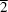 22 & 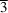 13, located at 17.73, 19.23 and 23.19 nm^−1^, respectively.

*In situ* WAXS measurements were performed during the CaCO_3_ precipitation reaction at the same times as in the SAXS experiments. The as-measured WAXS pattern principally shows the contribution of the scattering by water which dominates the signal (Amann-Winkel *et al.*, 2016 ▸). As shown in Fig. 8 ▸ (top panel, grey line), there is no clear sign in the scattering curve of any crystallized particles for the 9 m*M* solution, but only the signature of water. However, a careful subtraction of the water capillary contribution made on a series of more than 30 accumulated datasets measured at different times (see Fig. 8 ▸ top panel for the evolution of the WAXS datasets at indicated times) reveals that some very faint peaks with the same location as those of the powder collected after centrifugation of the same solution can be observed. At *t* = 106 s, there is no obvious sign of any peak but just some broad humps that could be attributed to ACC. At *t* = 136 s, some faint peaks appear. They drastically grow in intensity between *t* = 136 s and *t* = 166 s in a similar way to the Porod invariant. Their integrated intensity (shown in Fig. S3) mimics the evolution of the Porod invariant. The peak positions are the same as those of the dried particles (see Fig. 8 ▸ bottom panel). They are consistent with the vaterite polymorph (Christy, 2017 ▸; Cherkas *et al.*, 2018 ▸). We have used the *MAUD* software to calculate the X-ray powder diffraction pattern of the dried particles shown in Fig. 8 ▸ (Lutterotti *et al.*, 1999 ▸). The dataset was refined according to the monoclinic crystal structure of vaterite with the space group *C*2/*c* (Mugnaioli *et al.*, 2012 ▸). The refinement yielded the following lattice parameters: *a* = 1.2427 nm, *b* = 0.7133 nm, *c* = 0.9460 nm and β = 116.22°, which are very close to the values published by Mugnaioli *et al.* (2012 ▸). This resulted in good agreement between the experimetal and calculated diffraction profiles with *R*_wp_ = 5.25%. As the particles in the solution are rather small and due to the low precursor concentration (a resultant 4.5 m*M* CaCO_3_ concentration after mixing), the number of crystallized particles in the flow passing through the capillary was small. Given the large contribution of the water to the scattering, the signal of the crystalline particles was buried under this signal. This explains why the scattering by the particles is not observable unless the contribution of water has been subtracted accurately.

We can compare our results with those obtained by Bots *et al.* (2012 ▸) in terms of crystallization and particle size. They found that, about 1 min after mixing, ACC transforms into vaterite, and after 35 min, the particles have a diameter of 60 nm. They monitored the pH during the precipiation process and started at pH = 9.5, more acidic than in these experiments (pH = 11) and additionally a PSS additive was used. Our particles are clearly larger, probably because of the presence of PSS inside. Rodriguez-Blanco *et al.* (2008 ▸) also did not observe the formation of vaterite before at least 14 min of reaction at 7.5°C at a precursor concentration of 1 *M*.

### 4.4. Scanning electron microscopy imaging

**Figure 9:**
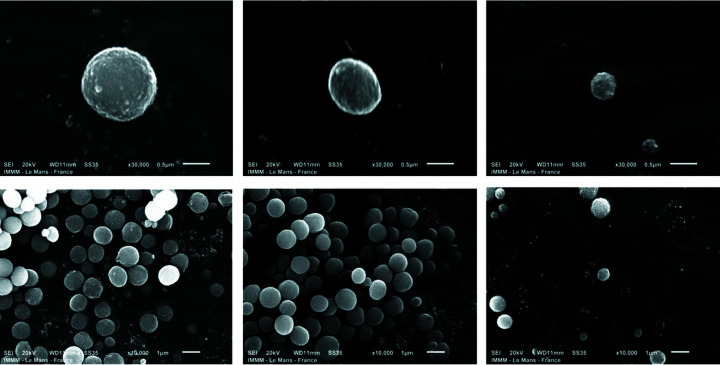
SEM images of the CaCO_3_ particles collected after centrifugation with varying initial concentrations of the precursors (left to right: *c* = 4.5 m*M*, 7 m*M* and 9 m*M*).

Samples prepared under similar conditions as for the SAXS/WAXS measurements were imaged by scanning electron microscopy after centrifugation. There is clear evidence of a decrease in the particle size as the concentration was increased. It can be seen from the SEM images shown in Fig. 9 ▸ that the particles are polydisperse and decrease in size when the precursor concentration is increased.

## 5. Conclusions

In this paper, we have reported the analysis of precipitation of CaCO_3_ particles as a function of time in the dilute regime using PSS as an additive. PSS considerably delays the precipitation of the particles with typical times above 60 s. We show that the use of the Kratky plot can provide extremely helpful information about the kinetics of formation of these particles. In particular, we demonstrate that the Porod invariant can be used to determine the scale factor of the scattered intensity, leaving the particle size distribution as the only function to be determined. The evolution of the Porod constant is analysed via an algebraic function of the sigmoidal type, with a characteristic time which depends on the initial concentration of the precursors. The evolution of the Porod invariant as a function of time shows three different stages among which we have identified stage 1 as the formation of clusters of PSS and the nucleation of nanograins, stage 2 as the growth by surface attachment of the nanograins, and stage 3 as the stationary state of the particles formed from attached nanograins. We further noted that, as the initial concentration of the precursor increased, the particle size decreased at constant PSS concentration.

The scattering profiles were further analysed using the Hurd–Flower model because the *q* dependence differs from the 1/*q* ^4^ behaviour characteristic of smooth-surface particles. The density autocorrelation function is characterized by *D*_f_ < 3 with a typical value *D*_f_ = 2.955. This dimension is compatible with the formation of particles composed of attached nanograins.

The WAXS experiments carried out at the same time as the SAXS experiments reveal the presence of amorphous particles of CaCO_3_ at *t* < 150 s which become crystallized after *t* = 150 s.

The evolution of the integrated intensity of the most intense Bragg reflection with time is somewhat similar to that of the Porod constant.

## Supplementary Material

Supporting figures. DOI: 10.1107/S1600576723005356/ge5135sup1.pdf
