# Supplementary material for: Insights into the precipitation kinetics of CaCO3 particles in the presence of polystyrene sulfonate using in situ small-angle X-ray scattering
Source: J Appl Crystallogr. 2023 Jul 14;56(Pt 4):1114–24. doi: 10.1107/S1600576723005356 (PMC10405600; doi:10.1107/S1600576723005356)
Supplement: Supplementary file 1 [file j-56-01114-sup1.pdf]

JOURNAL OF APPLIED CRYSTALLOGRAPHY

Volume 56 (2023)

Supporting information for article:

Insights into the precipitation kinetics of CaCO3 particles in the presence of polystyrene sulfonate using *in situ* small angle X-ray scattering

A. Gibaud, D. Younas, L. Mathews, T. Naranayan, K. Longkaew, I. U. Hageberg, Y. Chushkin, D. W. Breiby and B. Chattopadhyay

S1: Experimental set-up used to carry out the flow experiments

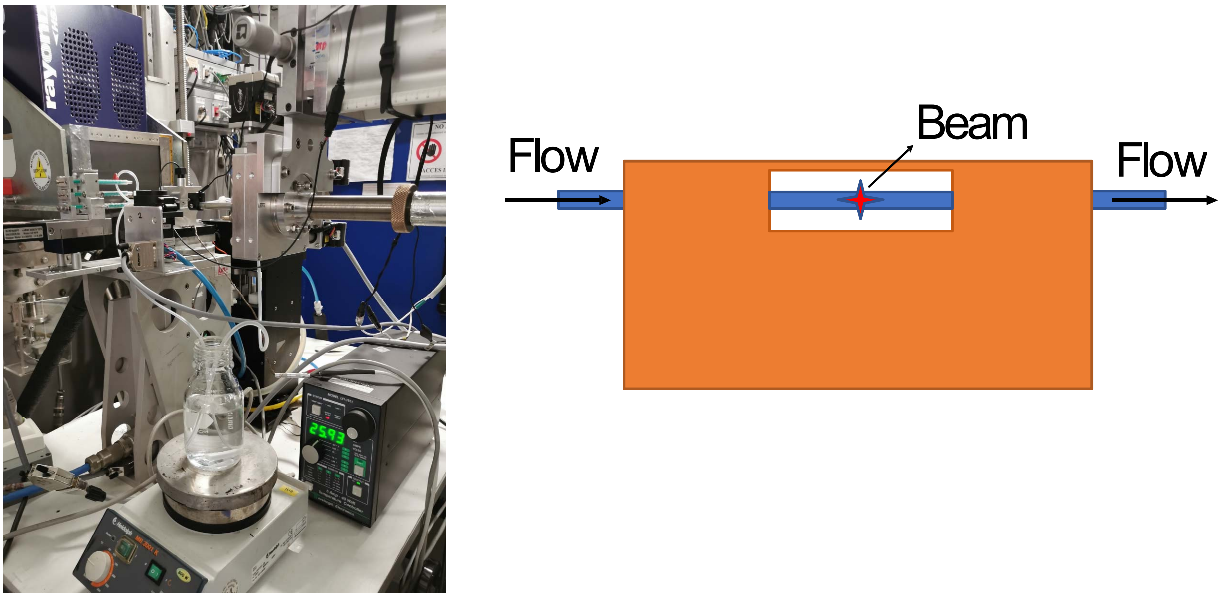

Fig. S1. A picture of the experimental set up at ID02 with, on the left, the flow through cell connected to the temperature controlled solution inside the beaker shown in the front and on the right a schematic representation of the sample cell.

S2 : Small Angle Scattering (SAS) Theory

Scattering by spherical objects

The scattered intensity of a spherical object is given by the modulus square of the scattered amplitude. The amplitude is the Fourier transform of the electron density. When the intensity is normalised to unity (see Fig. S2a), it corresponds to the modulus of the normalized form factor of this sphere and is given by

$$P(q, R) = \left(3\,\frac{\sin(qR) - qR\cos(qR)}{(qR)^3}\right)^2 \qquad (1)$$

Note that this quantity is dimensionless.

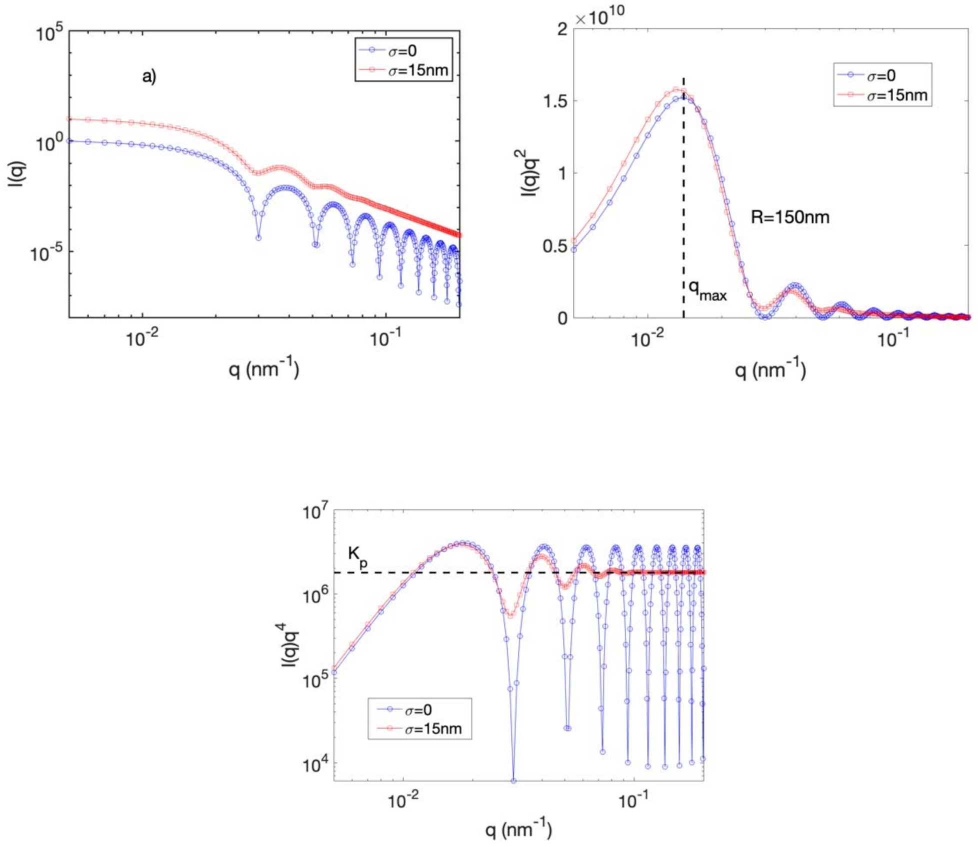

Figure S2: a) The intensity of a sphere of radius 150 nm (in blue) and with a gaussian distribution of radii with σ= 15 nm (in red). Intensity calculated from equation (4) was normalized to one. An offset of one order of magnitude between the two curves was introduced for clarity.
b) Kratky plot for monodisperse and polydisperse spherical particles calculated after Figure 1a). c) $I(q)q^4$ plotted versus q showing how to obtain the Porod constant K_p.

When the intensity is not normalized, the intensity has to be multiplied by a pre-factor depending on the volume of the sphere and by the contrast of electron density between the sphere and the solvent in which the sphere is suspended. The scattered intensity becomes

$$I(q,R) = r_e^2 \frac{V_p^2(\rho-\rho_{Sol})^2 P(q,R)}{V} = r_e^2 \left(\frac{4\pi R^3}{3}\right)^2 \frac{(\rho-\rho_{Sol})^2}{V} \left(3\,\frac{\sin(qR)-qR\cos(qR)}{(qR)^3}\right)^2 \quad (2)$$

where V and V_p are the irradiated volume and the volume of the particle respectively and r_e=2.810^{-15}m is the classical radius of the electron.

S3 : Integrated intensity of the most intense Bragg reflection located at 19.3 nm^{-1} as a function of time

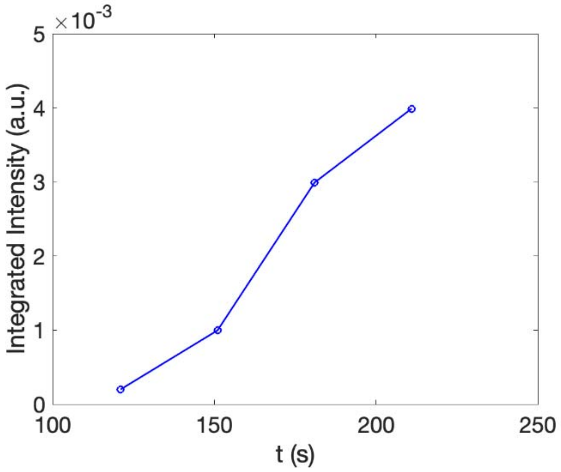

The integrated intensity of the most intense Bragg reflection located at q=19.3 nm^{-1} shown in the top part of Figure 8 is in fair agreement with the evolution of the Porod invariant shown in Figure 2. The data points correspond to the measured peak at the time given in Figure 8. Note that the very low intensity of the WAXS data sets impedes a better representation of this evolution as it is necessary to sum 30 frames to obtain a reasonable statistics in these plots.
